# Recent progress in the study of transition in the hypersonic boundary layer

**DOI:** 10.1093/nsr/nwy052

**Published:** 2018-05-07

**Authors:** Cunbiao Lee, Shiyi Chen

**Affiliations:** State Key Laboratory for Turbulence and Complex Systems, Advanced Aero Engine Collaborative Innovation Center, Peking University, Beijing 100871, China

**Keywords:** hypersonic boundary layer, transition, quiet wind tunnel

## Abstract

Turbulence is a universal form of fluid motion. It is the key issue in fluid mechanics. Very recently, it has become a bottleneck in some key engineering research of national importance, such as aeronautics, astronautics and navigation. Developed turbulence and the onset of turbulence, i.e. transition, are two interrelated parts of turbulence. The hypersonic boundary-layer transition is a strategic focus in the fluid mechanics community. This article reviews recent developments in the study of the hypersonic boundary-layer transition, research facilities and experimental techniques. The hypersonic quiet wind tunnel is introduced as a necessary device to obtain real flight data in near space. Near-wall measurement techniques, such as temperature-sensitive paint, near-wall particle image velocimetry and Rayleigh-scattering visualization, are shown. The most important issues in the recent development of the transition in the hypersonic boundary layer are addressed. The instability and nonlinear interaction of different instability modes are discussed. The recent contributions from China, especially at Peking University, are also introduced.

## INTRODUCTION

The stability of the boundary layer and its transition to turbulence have been studied for more than a century. Reviews of this research have been given by Reshotko [1–4], Morkovin and Reshotko [5], Kachanov [6], Saric *et al*. [7–9], Horvath *et al*. [10] and Lee and Wu [11]. Stetson and Kimmel [12], Schneider [13–15], Fedorov [16] and Zhong [17] have provided useful summaries and discussions about hypersonic boundary layers. A unique feature of a hypersonic boundary layer is the presence of a family of instability modes, the Mack modes. In a low-speed boundary layer, there exists only one instability mode, Tollmien–Schlichting (T–S) waves, while in a hypersonic boundary layer, besides the first-mode instability, which is the counterpart of T–S waves in the low-speed boundary layer, there exist higher unstable modes. The so-called Mack modes consist of these linear unstable modes. In addition, they contribute to the transition with large-amplitude fluctuations. These make the trends at low speed different from those of hypersonic regions, such as the effect of surface temperature.

Experimental works that investigate not only the location of transition but also the mechanisms involved are essential to the improvements of modern theories. Accordingly, relevant experimental studies are indispensable for at least two reasons. Firstly, the key mechanisms need to be identified in part through experimental work. Secondly, the key numerical assumptions and results need to be validated experimentally. Most of the ground-test experiments are conducted in conventional wind tunnels with much higher disturbance levels than those in flight. The flight transitional Reynolds number can be obtained by a facility called a quiet wind tunnel [15,18–20]. There are only four of these quiet wind tunnels in the world at present with a speed of Mach 6 and a moderate Reynolds number, which are achieved by a laminarized boundary layer in the nozzle wall and operated in cold flow.

**Figure 1.**
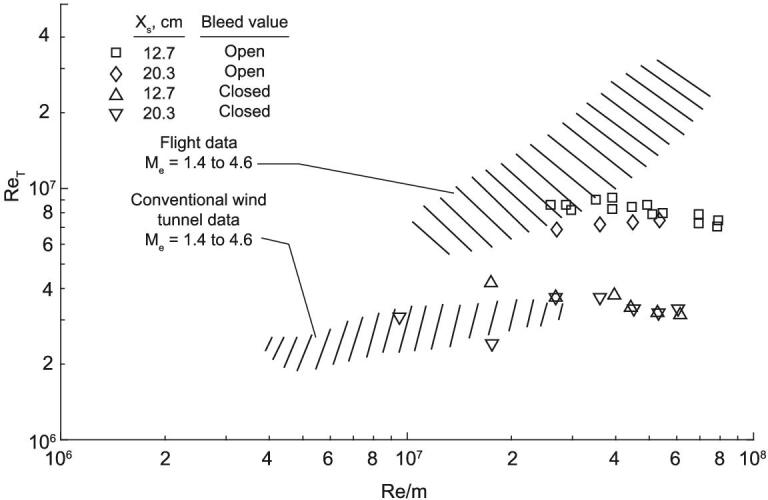
Variation of transition Reynolds number on sharp-tip cones with experimental data in the Mach-3.5 tunnel under partially quiet flow. The square symbols show the data obtained in a noisier environment while the triangle symbols show data from a quieter environment. Reprinted with permission from [21]. Copyright American Institute of Aeronautics and Astronautics.

Due to the strategic importance and great potential of the near-space region, development of hypersonic vehicles for near space has become a hot topic for military research. As a key to the design of hypersonic vehicles, the process of the hypersonic boundary-layer transition presents a number of scientific challenges that are still to be investigated. A profound understanding of the mechanism of the hypersonic boundary layer is crucial to the design of thermal protection systems and flight control. As known to all, the real flight environment is several tens of kilometers above the ground where the freestream is rather quiet. On the other hand, conventional wind tunnels suffer from high level of freestream fluctuation, which is of the order of one to two above that of the flight environment. The fluctuations dominated by acoustic noise radiated from the turbulent boundary layers on the nozzle walls have dramatic effects on the boundary-layer transition process of the downstream models and degrade the wind tunnel experiment results to a great extent. For example, for a flat-plate model the transition Reynolds number acquired in conventional wind tunnels is one order lower than that in quiet-flow wind tunnels. Figure 1 shows the transition Reynolds numbers obtained in conventional wind tunnels, quiet-flow wind tunnels and real flight environments, respectively. It can be seen that the conventional wind tunnel results are very different to the flight data [21]. In addition, various models might differ in their sensitivity to the large fluctuations of the freestream. It is difficult to perform effective experiments on the hypersonic boundary-layer transition in conventional wind tunnels.

## FACILITY: ROLE OF THE QUIET WIND TUNNEL

Quiet-flow wind tunnels provide low noise levels that are comparable to real flight. Because the transitional Reynolds number predicted in quiet wind tunnel is the same as in real flight, the wind tunnel is considered as part of the national strategic equipment. It is used to predict the flow transition, which is direct related to aerodynamic heating. Accurate evaluation of aerodynamic heating is a key issue in hypersonic flight vehicle design. The construction of a hypersonic wind tunnel is a national strategy. It is not easy to develop comparable low-noise facilities at hypersonic flows, for several reasons. First, the transducer for measuring disturbance is a challenge due to the high level of pressures, temperatures, and disturbance frequencies in hypersonic flow. Second, the acoustic waves scattered from the nozzle walls are the dominant source of noise. Reduction of the noise level is the main focus in quiet tunnel design. Third, matching experimental techniques are very difficult and need to be developed independently.

Since the 1990s, various types of research on hypersonic quiet wind tunnels have been carried out in China (while pioneering research has been carried out abroad since the 1960s). Zhou and Chang were the first researchers in China to study quiet-flow wind tunnels [22–24]. They began theoretical and experimental research on hypersonic quiet wind tunnels in1997. With the support of a national defense study-in-advance and other resources, a low-noise Ma 4.0 wind tunnel with a nozzle 120 mm in exit diameter was built in 2000. However, due to the limitations of funding, manufacturing techniques and materials, quiet-flow conditions achieved by the wind tunnel were limited. The maximum freestream Reynolds number under quiet-flow conditions was about 1.0 × 10^6^ m^-1^, compared to 10^7^ m^-1^ in-flight. Nevertheless, the wind tunnel provided valuable experience and a reference for the future development of quiet wind tunnels in China.

**Figure 2.**
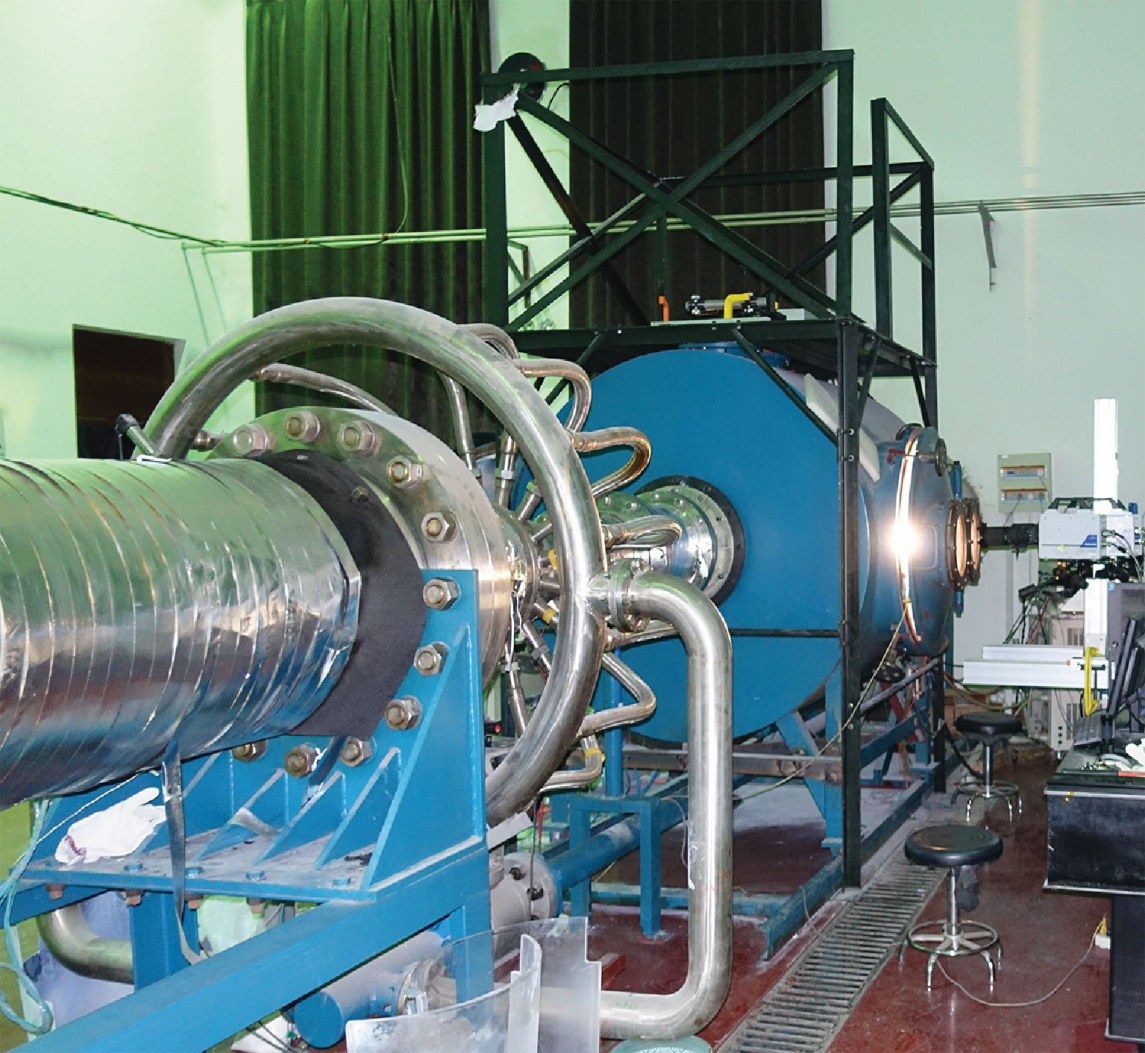
Hypersonic wind tunnel of Ma 6.0 and D300 in the nozzle.

Between 2010 and 2011, Peking University (PKU) built a low-noise Ma 6.0 wind tunnel with a nozzle 300 mm in exit diameter (Fig. 2). Calibration results showed that the wind tunnel operated in quiet conditions with a stagnation pressure less than 0.25 MPa. The total temperature was 430 K. The noise level rose rapidly when the stagnation was higher than 0.25 MPa. The maximum freestream Reynolds number under quiet-flow conditions was about 2.5 × 10^6^ m^-1^ [25–27]. In recent years, in order to increase the maximum freestream Reynolds number under quiet-flow conditions so that the wind tunnel condition could be comparable to the flight environment, Lee and Zhou drew on the experience of Beckwith and Schneider [25–27].

With the innovations in design and manufacturing methods, the State Key Laboratory of Turbulence and Complex Systems at Peking University developed a series of nozzles 300 mm in exit diameter covering both supersonic and hypersonic Mach numbers (Ma 3.0, 4.0, 5.0, 5.5, 6.0, 6.5).These nozzles are by far the largest nozzles in operation in the world.

There are three quiet hypersonic wind tunnels in operation around the world, as listed in Table 1.

**Table 1.** Quiet hypersonic wind tunnels in operation.

Location	Nominal Mach number	Diameter of nozzle exit (mm)	Maximum Re_unit_ under quiet conditions (m^-1^)	Run time (s)	Disturbance level *p*′/*p*_mean_
Purdue University	6.0	241	1.1 × 10^7^	7 ∼ 8	<0.05%
Texas A&M University	5.9	191	0.46 ∼ 1.1 × 10^7^	up to 40	<0.08%
Peking University	6.0	120	1.0 × 10^7^	up to 50	<0.2%
		160	1.0 × 10^7^		<0.2%
		300	without permission		without permission
	3	300	without permission		without permission
	4				
	5.5				
	6.5				

The pressure transducers manufactured by Kulite Company were used by Beckwith [28] to measure the Pitot pressure fluctuation in order to determine the fluctuation level. Kulite XCQ-062–25A pressure transducers were used to measure the fluctuation level in Peking University's Ma 6.0 Φ180 mm wind tunnel.

**Figure 3.**
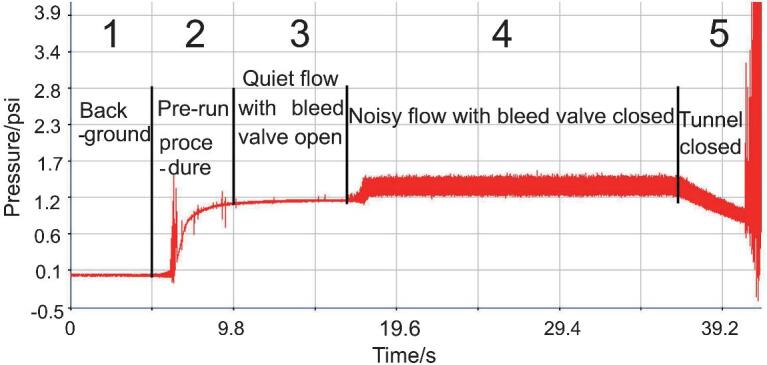
Kulite Pitot pressure trace for Ma 6.0. The total pressure was measured for both quiet and noisy flows in one run. Stage 1 measures the pulsing signal when before a run of the tunnel, which represents the electrical noise level. During Stage 3, the wind tunnel runs under quiet conditions; it can be seen that the pulse of total pressure is of the same level as in Stage 1. During Stage 4, the wind tunnel runs under noisy conditions. The amplitude of pulses is significantly larger than that under quiet conditions. Reprinted with permission from [29]. Copyright Springer Nature.

The transducer was aligned in the centerline of the nozzle. The time series of the Pitot pressure fluctuation measurement is shown in Fig. 3 [29]. Different flow states are denoted by numbers in the figure: State 1 denotes the background fluctuation before the start of the wind tunnel; State 2 denotes the pre-run period when the flow field is being established; State 3 denotes the pressure fluctuation when the suction valve is open and the flow is quiet; State 4 denotes the noisy flow when the suction valve is closed; and State 5 denotes the shut-down of the wind tunnel.

The noise level of a wind tunnel is defined by the RMS value of the Pitot pressure fluctuation *p*′ divided by the average pressure *p*_mean_. As shown by the signals in State 3, when the flow is quiet, the fluctuations around the average pressure are within the background noise level, giving a noise level less than 0.2% and a Reynolds number of about 10^7^ m^-1^ for PKU's Φ180 mm quiet wind tunnel. For a Φ 300 mm quiet wind tunnel, the noise level is low enough and the Reynolds number is high, and the real experimental Reynolds number of the test mode is the same as that at Purdue University. The quiet tunnel offers more opportunities for both fundamental types of research and industrial applications in China.

## IMPORTANT EXPERIMENTAL TECHNIQUES

### Temperature-sensitive paint (TSP)

Temperature-sensitive paint is a powerful technique that is used to predict transition. It can offer all the surface transition information of the flight vehicle. The development of TSP has attracted a great deal of attention worldwide. TSP is the application of a special kind of fluorescent molecules. These molecules emit another wavelength of fluorescence when inspired by a laser beam of specific wavelength. The quantum efficiency of the luminescence decreases with increasing temperature; this effect associated with temperature is thermal quenching, which serves as the major working mechanism for TSP [30–33].

The fluorescent molecules are painted on the surface of the experimental model. When the fluorescent molecules are inspired by light of a specific wavelength, the full field temperature information can be obtained using charge coupled device (CCD).

**Figure 4.**
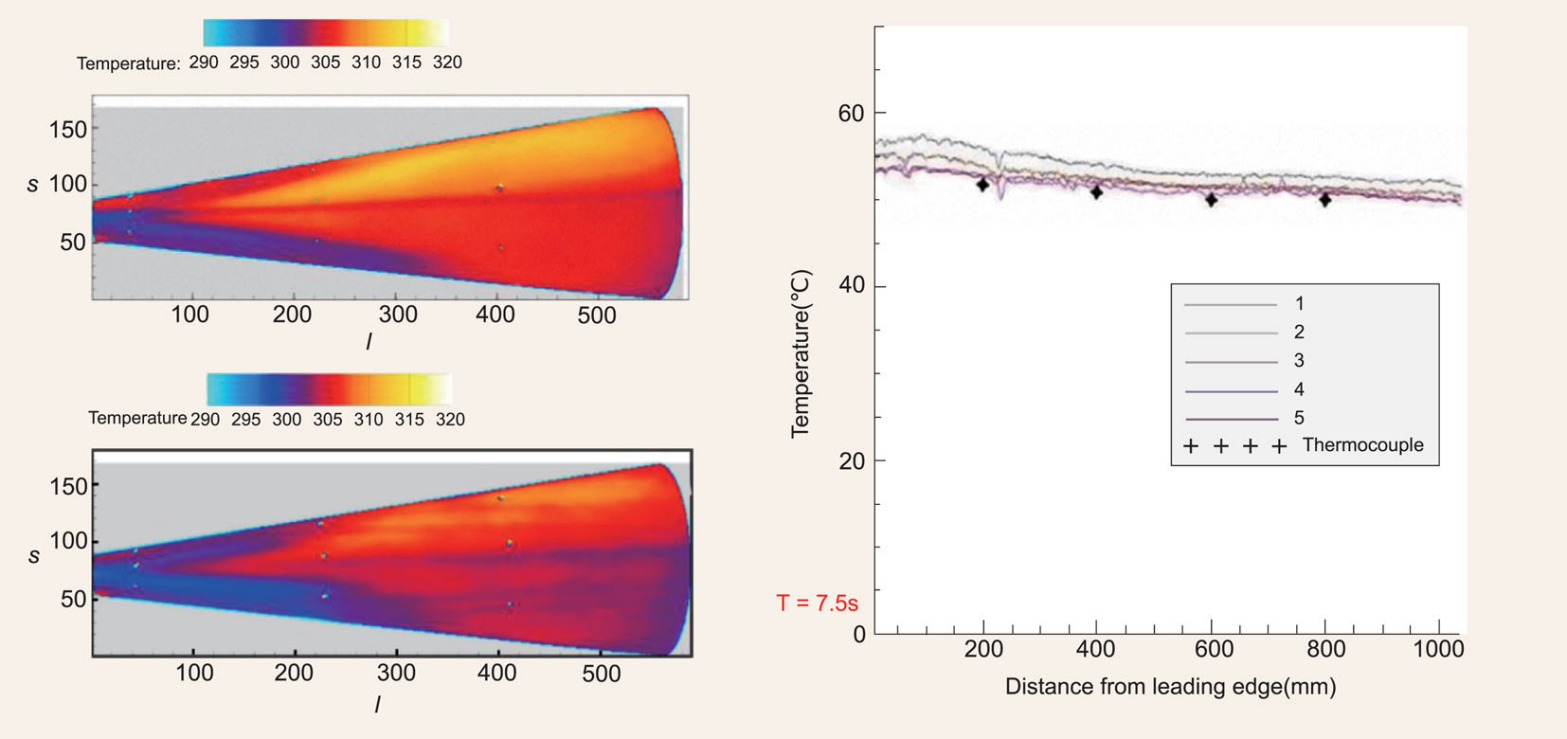
Windward (top left) and leeward (bottom left) temperature distribution by TSP over a Ma 6 straight cone at a 5° angle of attack with their comparisons to thermal couples (right).

Successful aerodynamic experiments have been undertaken at NASA Arnold Center and NASA Langley Center using the TSP produced by Purdue University. Very recently, TSP was used by Ward *et al*. [34] and Liu *et al*. [35] to measure the temperature and the heat transfer in hypersonic flows in the BAM6QT quiet tunnel. The data measured by TSP and by heat transfer transducer are in good agreement, as shown in Fig. 4. This means that the quantitative application of TSP in hypersonic flow is successful at PKU.

**Figure 5.**
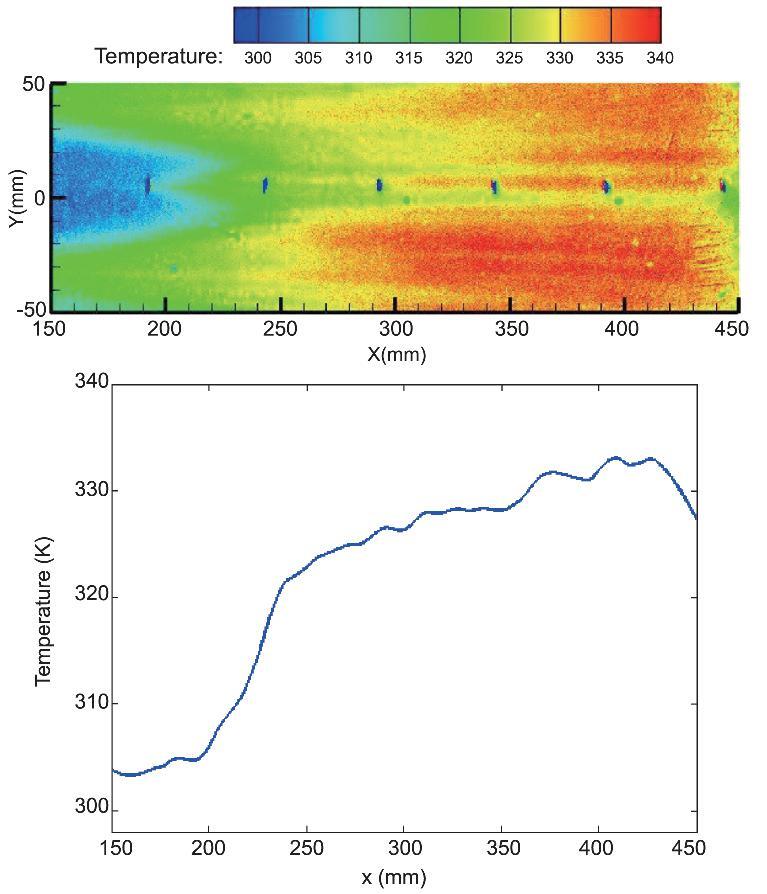
Temperature distribution over a hypersonic flat plate by TSP (top) and the evolution of the temperature along *y =* 12 mm (bottom).

Figure 5 shows a temperature distribution over a hypersonic flat plate. The transfer caused by skin friction increases as high as three times that before transition. As a result, the surface temperature, measured by TSP, experiences a sharp increase at the transition location.

**Figure 6.**
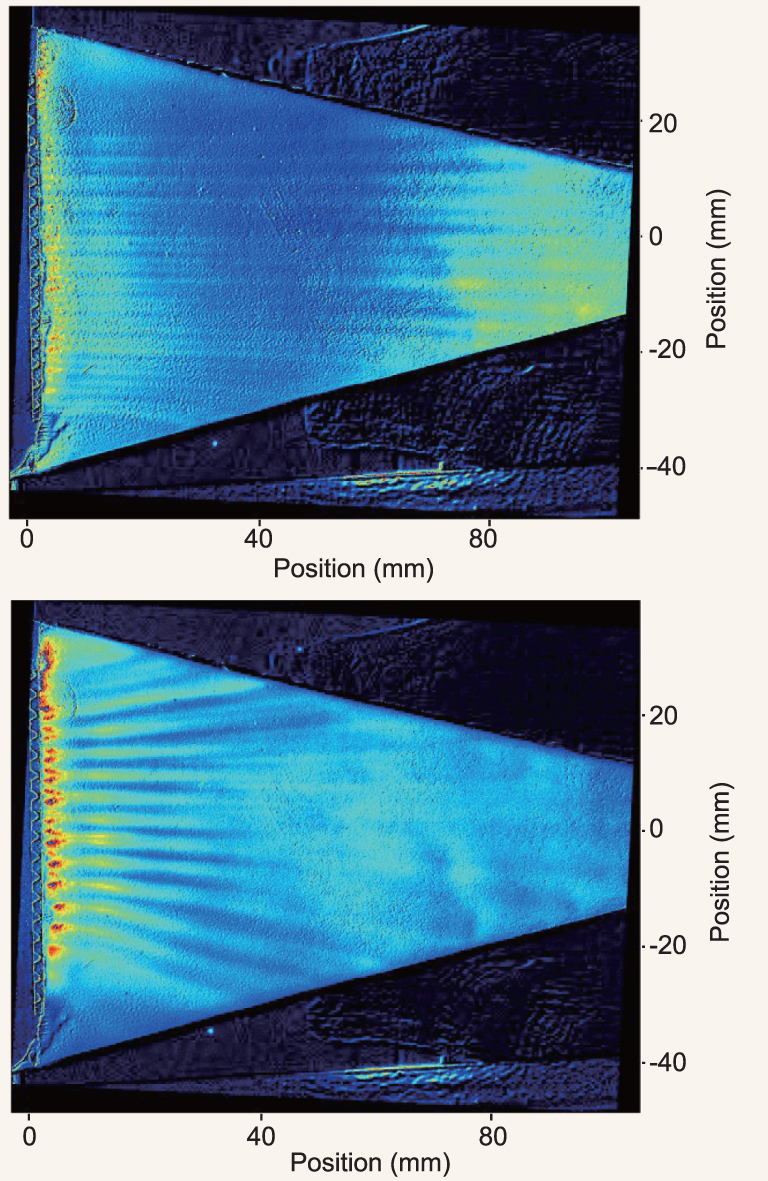
Comparison of temperature distribution over a windward concave surface of a hypersonic model between noisy (top) and quiet (bottom) conditions.

Figure 6 shows the temperature distribution over an air-inlet model for the scramjet configuration under noisy and quiet conditions in the hypersonic wind tunnel. Streamwise hot streaks, which are related to both the effects of leading-edge roughness and Görtler instability, are observed over the surface. Unlike noisy conditions, a cross-flow effect exists under quiet conditions, which will inevitably decrease the air intake quantity of the configuration. Note that the quiet conditions approach the real flight conditions.

### Near-wall PIV technique for hypersonic flows

Particle image velocimetry (PIV) is a vital technique for real-time measurement of the flow field of a plane or a volume. With the rapid improvements of CCD cameras, PIV is now commonly used to study high-speed flows. The key issues in PIV technology include the selection of seeding particles, particle dispersion in the flow, and analysis of particle image recordings. The uncertainties in near-wall PIV mainly arise from the analysis algorithm, particle dispersion, and signal-to-noise ratio of particle images. The algorithm has been discussed by Zhu *et al*. [36], who proposed a preprocessing method for recorded images in which particles undergo a large in-plane displacement and large velocity gradient.

**Figure 7.**
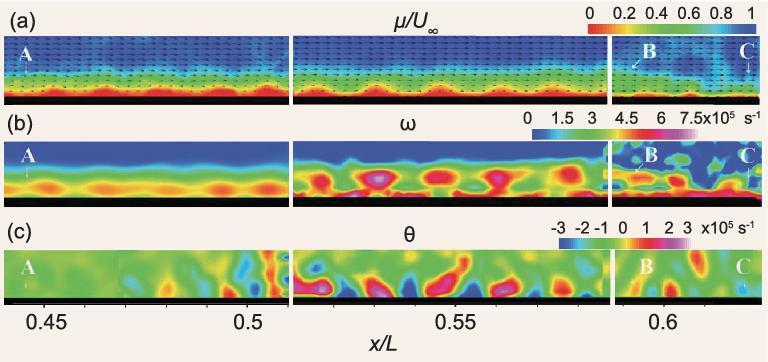
PIV results of boundary-layer development over a Ma 6 flare cone on a velocity field (a) with its vorticity (b) and dilatation (c). Reprinted with permission from [37]. Copyright American Institute of Aeronautics and Astronautics.

Figure 7 shows the first PIV measurements of the entire evolution of the transition process over a Ma 6 flared cone. The second-mode evolution with its strong dilatation process and modification on the vorticity field are presented. The local velocity profile, with its mean and fluctuated parts, shows good agreement with the calculations based on linear stability theory [37]. So far, only a few research groups in the world have the abilities to measure near-wall flow structures by the means of PIV in hypersonic flows [36–38]. A 2D second-mode measurement very near to the wall was first obtained at PKU. The influence of varying 3D, cylindrical post-type trip size on the mean and turbulent velocity profiles of Mach 7.6 was examined using PIV at Princeton University and the University of Washington. Tomographic PIV was applied for the first time in a boundary-layer transition of Mach 7.5 at Delft University and the University of Naples. The spatial information obtained by PIV gives a deep insight into the transition of hypersonic flows.

### Rayleigh-scattering flow visualization

**Figure 8.**
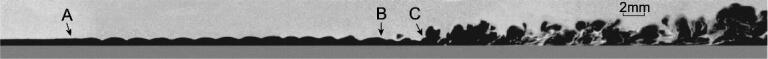
Rayleigh-scattering visualization of the transition stages. Reprinted with permission from [50]. Copyright American Institute of Aeronautics and Astronautics.

An instantaneous and nonintrusive technique is vital in measuring the flow field. In addition, pulsed laser-based measurement is widely applied in flow visualization. Most of these techniques require particle tracers that scatter light according to their size [39–42]. It is found that the scattering in the Rayleigh-scattering technique is predominantly caused by clusters that are formed as a result of the cooling effect during the expansion process [43,44]. An investigation of the Rayleigh signal in such an expansion indicated that it strongly depends on the local values of pressure and temperature [45,46]. By injecting carbon dioxide (CO_2_) with a purity of 99.99% into the tunnel from upstream of the electric heater, the structure of the flow could be visualized owing to the condensation process as the carbon dioxide passed through the test section with a static temperature lower than 70 K. The diameter of condensed particles is much smaller than the wavelength of the laser, leading to Rayleigh scattering. The temperature is high near the wall, which makes the solidified carbon dioxide become gas. The interface of carbon dioxide with different kinds of state is shown as a condensation line between black and white parts in a recording picture, which could represent the flow structure when it was disturbed by instability waves in the boundary layer. This technique was applied by Smits' group to investigate different kinds of hypersonic boundary layers [47–49]. Figure 8 shows the whole evolution of the transitional process in the hypersonic boundary layers [50]. Unlike incompressible flow, second-mode patterns are generated by the linear instability of the hypersonic flow. The second-mode waves are weak at the early stage in the region between arrows A and B, but they develop into a turbulent state after arrow C. The regular rope-like structures of the early stage are second-mode waves with a wavelength of about 2 mm. They persist for a long distance and then decay into a region of small amplitude between arrows B and C, finally becoming a turbulent boundary layer on the right. Such a clear and complete visualization of the transition process has not been reported before.

## TRANSITIONAL MECHANISM OF THE HYPERSONIC BOUNDARY LAYER

The study of laminar–turbulent transition in hypersonic boundary layers is of great importance to the thermal protection systems and aerodynamic design of vehicles that travel in near space at sustained hypersonic speeds. As is commonly accepted, since most near-space hypersonic flights confront a quiet freestream with small disturbances, the path of transition can be broadly divided into three stages: (i) receptivity, (ii) linear eigenmode growth or transient growth, and (iii) nonlinear breakdown to turbulence.

Receptivity is the first stage in which environmental disturbances are ‘sensed' by the boundary layer and converted into the initial instability waves (such as first-mode or second-mode instability waves) in the boundary layer.

During the second stage, the boundary-layer instability waves undergo linear eigenmode growth, which can be derived according to linear stability theory (LST) by solving the homogeneous linearized disturbance equations. Besides the Mack modes, the Görtler mode may undergo considerable growth in certain cases (e.g. along with a concave surface). Unlike the Mach mode, the Görtler mode is a centrifugal instability in nature and can be either steady or unsteady depending upon the steady instability [51,52].

As the instability waves grow to certain amplitudes, secondary instability or other nonlinear interactions begin to take effect and eventually lead to the nonlinear breakdown to turbulence. There exist exceptions called the bypass transition where the receptivity to large-amplitude freestream disturbances bypasses the second stage and leads to a transition.

The basic process of transition in an incompressible boundary layer has been discussed in detail by Lee and Wu [11]. Based on recent progress in the study of transition in the hypersonic boundary layer [37,50,53], a similar transition scenario for the hypersonic boundary layer is proposed in the conclusion.

### Receptivity

As mentioned above, the receptivity process determines the initial flow conditions, which strongly affect the transition process. The receptivity processes in the compressible boundary layer have been extensively studied and include freestream disturbances (acoustic, vortical, and thermal perturbations) and wall-induced disturbances (roughness element, vibrations, periodic suction/blowing, and surface heating); we refer the readers to [16,17,54].

**Figure 9.**
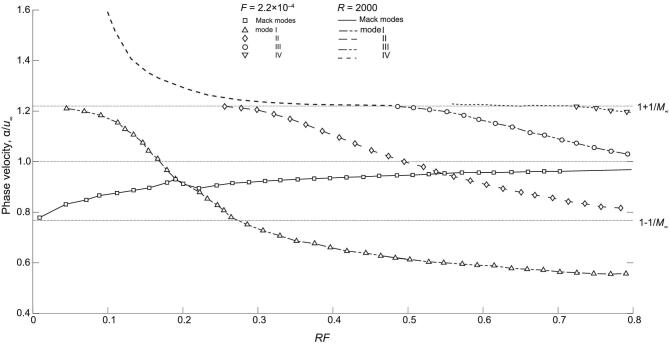
The distribution of the phase velocities of boundary-layer wave modes as a function of RF obtained by LST. Reprinted with permission from [55]. Copyright Cambridge University Press.

In general, there are two families of modes in hypersonic boundary layers, namely Mack modes (the first mode and second mode) and stable modes (Mode I, Mode II, etc.); see Fig. 9 [55]. The linearly stable modes are especially important in receptivity studies because they could have resonant interactions with both forcing waves and Mack modes and transfer the energy from the former to the latter.

The details of the recent achievements in receptivity are given in [16,17]. Jiang and Lee gave a summary in Chinese that described the freestream disturbances and wall-induced disturbances [56].

In fact, the linear stability theory just shows the possibility that the disturbance can generate instability, create flow structure and produce turbulence. The key problem of the instability experiments is how to select the disturbance. Kachanov and several other groups have produced turbulence using an artificial technique predicted by the linear stability theory [6].

According to linear stability theory (LST), in a hypersonic boundary layer, besides the first-mode instability there are higher unstable modes, the frequencies of which are much higher than that of the first mode. Among these modes, the second mode has received the most interest in the study of the hypersonic boundary layer. The largest amplification rate of the second-mode instability begins to exceed that of the first mode as Mach number reaches about 4 [57].

**Figure 10.**
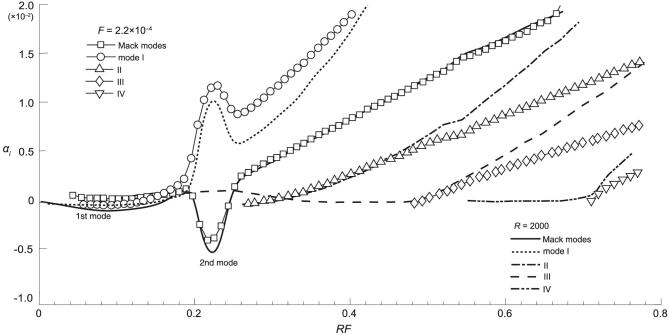
Space amplification rate predicted by LST (R refers to the normalized location and F refers to normalized frequency). Reprinted with permission from [55]. Copyright Cambridge University Press.

As shown in Fig. 10, for a first- or second-mode unstable wave, given a geometry and location, LST predicts the space amplification rate for a certain frequency [55]. Therefore, the frequency of the most likely excited disturbance wave can be chosen in experiments according to the LST prediction in two possible ways: For a given mode and measurement location χ_1_, one can choose the frequency with the largest local space amplification rate *α_i_* at *χ_1_*. Alternatively, one calculates the so-called *N*-factor $(N = \int_{{{x_0}}}^{{{x_1}}}{{{\alpha _i}dx}})$ by integrating the predicted space amplification rate along the streamline from a certain location ${x_o}$, upstream of ${x_1}$, and chooses the frequency with the largest *N*-factor. The former method chooses the locally most amplified frequency. The latter method chooses the disturbance wave most amplified during a certain path along the streamline.

In the environment of a hypersonic wind tunnel (∼500 Pa), a stable glow discharge can be generated between two parallel electrodes loaded with high-voltage (∼2 kV) and high-frequency (20 kHz–200 kHz) current. Glow discharge introduces acoustic-nature disturbances into the freestream [58], which can be used as an artificial disturbance in the plate boundary layer.

Bountin published an empirical formula associating the local Reynolds number with the frequency of the second-mode waves that have the maximum growth rate [59]. Combining the empirical formula and the LST analysis, we found a possible frequency range to excite the strongest second-mode waves using glow discharge. LST was also used to study the frequency range of the first-mode waves with the maximum growth rate.

The experiments were carried out in the Ma 6.5 Φ300 mm hypersonic quiet wind tunnel at Peking University to verify the theoretical analysis and to determine the exact frequency. High-voltage AC power at different frequencies was tested to drive the electrodes on the surface of the plate model to discharge. The evolution of the artificial disturbance generated by the discharge was observed using the CO_2_ Rayleigh-scattering flow visualization technique and near-wall PIV technique. Experimental results demonstrated that at a frequency of about 105 kHz, the glow discharge can excite clear second-mode waves in the boundary layer. In addition, an interesting phenomenon was found that, when applying discharge at the first-mode frequency (∼30 kHz), notable growth of the second-mode waves could also be observed. More analysis and experiments are still going on to understand the influence of glow discharge on the boundary-layer transition.

Roughness, one of the typical wall-induced disturbances, can generate instability waves of both first mode and second mode. There are three major effects of roughness: the disturbance amplitude is controlled by its height; the frequency is controlled by its streamwise scale; and the waveform (2D or 3D) is determined by its geometric shape.

### Nonlinear interaction between modes

Three types of instabilities, i.e. first-mode, second-mode and Görtler-mode instabilities are very common in hypersonic boundary layers. The oblique component of the first-mode instability is mostly unstable, as is the planar component of the second-mode instability. The Görtler mode can be either steady or unsteady with the former usually being dominant. Many people have focused on the steady Görtler modes, while unsteady Görtler vortices have been studied only recently [51,52]. In generally, the frequencies of the first-mode waves are around tens of kilohertz, whereas the frequencies of the second-mode waves can be even ten times higher. Therefore the interactions between the second mode and the first mode/Görtler mode are important since they represent the interactions between very low-frequency and high-frequency waves and they can provide new spectrum-fulfilling mechanisms. Therefore, this topic has been theoretically and numerically studied by many people. In a numerical study, Maestrello *et al.* [60] have shown that the second mode can increase the nonlinearities of the flow field. Al-Salman [61] applied asymptotic analysis to study the interaction between a planar second-mode wave and one/a pair of oblique first-mode wave/s. He reveals that the first-mode waves will go through super-exponential growth due to phase-locked interactions with the second-mode waves. Whang and Zhong [62] performed a numerical simulation on interactions between steady Görtler vortices and second-mode waves. It is found that the inflectional regions formed by Görtler vortices may support second-mode waves. Also with direct numerical simulations, Yu and Luo [63] studied the interactions between a planar second-mode wave and a pair of first-mode waves, and found that the spanwise harmonics were heavily promoted. Using parabolized stability equations (PSE), Ren and Fu [64] have shown that finite-amplitude steady Görtler vortices can stabilize both first- and second-mode waves under certain conditions. However, the details of the interactions between the first- and second-mode waves, as well as the interactions between the second mode and steady/unsteady Görtler vortices, remain unclear.

In experiments, nonlinear interactions among various modes are often identified using bicoherence. In a Mach 8 experiment, Kimmel and Kendall [65] observed strong bicoherence between the low-frequency modes and the second mode. They attributed the low-frequency mode to the response of the boundary layer to ambient tunnel disturbances, which is also supported by Lachowicz *et al*. [66]. Later, strong interactions between low-frequency and second-mode waves were also reported by Chokani [67,68]. They performed a bicoherence analysis based on the hot-wire data obtained in a Mach 6 quiet tunnel. Their results indicated that the low-frequency modes can arise from sideband interactions of second-mode waves. For a Mach 6 boundary layer on a sharp cone, Shiplyuk *et al*. [69] identified the subharmonic resonance as well as interactions between the first- and second-mode waves. Hofferth *et al*. [70] performed focused schlieren measurements on a flared cone in a Mach 6 quiet wind tunnel. They also observed interactions between second-mode waves and low-frequency disturbances. Munoz *et al*. [71] conducted wall-pressure measurements along an inclined cone at Mach 6. They also identified the interactions between the low-frequency waves and the second-mode waves. Quite recently, Zhu *et al*. [37] (referred to here as the PKU experiments) found that the low-frequency waves (around 30 kHz) were amplified whereas the second-mode waves (around 350 kHz) were largely reduced before the transition.

**Figure 11.**
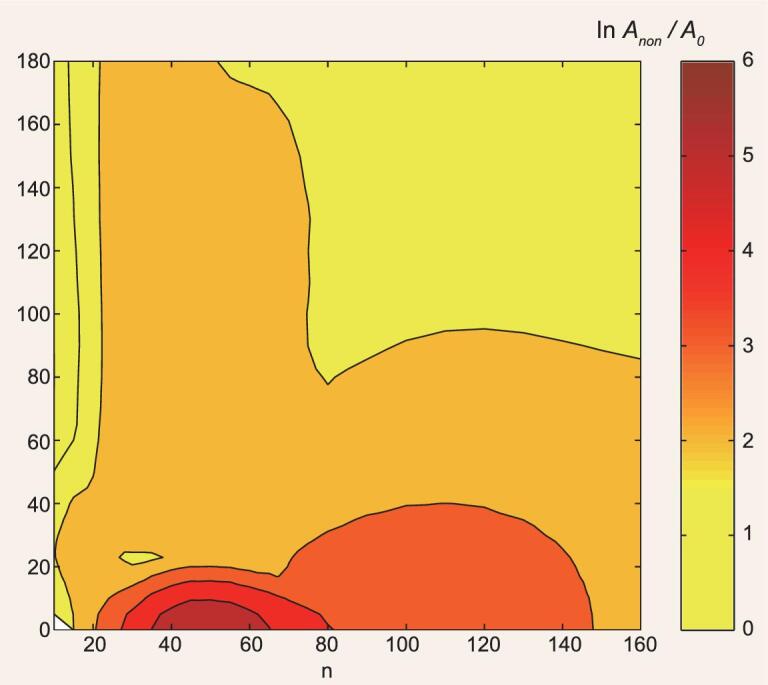
Contours of amplitudes spectra in the azimuthal wave number–frequency plane at *x =* 180 mm. Reprinted with permission from [72]. Copyright Cambridge University Press.

Chen *et al*. [72] have performed a detailed stability analysis of the boundary layer of a flared cone for the same flow conditions as in the PKU experiments. Using linear stability analysis and parabolized stability equations (PSE), Chen *et al*. [72] showed that the low-frequency waves likely arise from either the Görtler vortices or nonlinear forced waves from the sideband interactions of the second-mode wavepackets or the streamwise vortices driven by a pair of first-mode waves and that they interact with the second mode via the phase-locked mechanism and the subsequent parametric-resonance mechanism. The parametric study shows that very low-frequency modes (belonging to the unsteady Görtler vortices with frequencies below about 30 kHz and azimuthal wave numbers around 50) are most heavily promoted, as shown in Fig. 11. Chen *et al*. [72] classified the typical evolution of the low-frequency mode into the linear stage, the synchronization stage, the parametric-resonance stage and the full-interaction stage. At first, fast-growing difference and sum modes are generated and driven by interactions between the low-frequency mode and the second mode. These nonlinearly forced modes in turn make the low-frequency mode saturated or slightly attenuated. Meanwhile, the wave numbers of the low-frequency and difference/sum modes are rapidly adjusted in order that the resonance condition is approximately satisfied. The low-frequency mode then enters the parametric-resonance stage where it grows much faster than linear theory predicts. Finally, these modes become fully coupled. We note that the phase-locked theory can be applied in the linear stage since the phase velocities of the disturbances are nearly the same. However, at the parametric-resonance stage, the phase velocities of the disturbances become quite different so the phase-locked theory may not be suitable.

**Figure 12.**
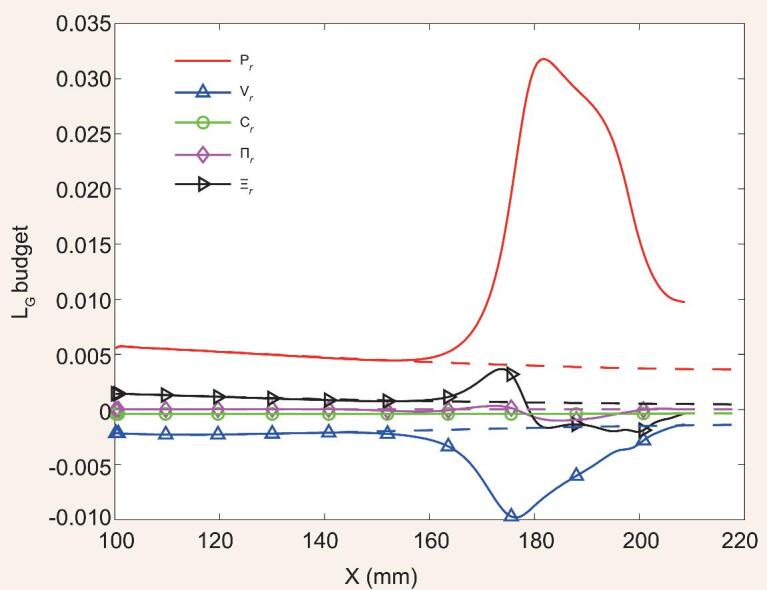
A linear energy budget for the low-frequency mode (frequency 20 kHz and azimuthal wave number 50). The five terms in the legend represent the production, viscous dissipation, curvature term, pressure dissipation and nonparallel term, respectively. Reprinted with permission from [72]. Copyright Cambridge University Press.

Energy method analysis further shows that the rapid growth of the low-frequency mode is solely due to the sharp increase of the Reynolds stress work, which is a linear mechanism, as shown in Fig. 12. In contrast, the rapid growth of the difference/sum modes and the rapid decay of the second mode are solely due to the work done by the nonlinear terms.

**Figure 13.**
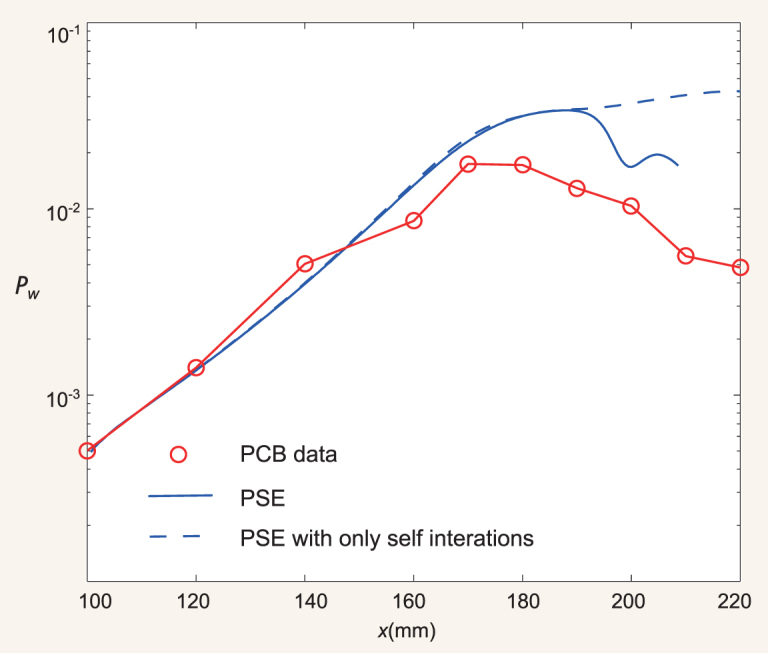
Comparison of the measured PCB data and the PSE predictions for the wall-pressure amplitudes of the second mode. The PCB data were derived from the RMS of the filtered time series with the passband (330 kHz, 370 kHz). The PSE was set so that its initial value is equal to the PCB data at this point for comparison. Reprinted with permission from [72]. Copyright Cambridge University Press.

The parametric study also reveals that the interactions between a second-mode wave and a single first-mode wave are not so strong. However, a spanwise mean-flow-distortion (MFD) can be created by a pair of first-mode waves. Consequently, the second mode, the spanwise MFD and the fundamental oblique wave can be involved in a resonance downstream, which makes the spanwise MFD and oblique wave increase sharply. The evolutions of the second-mode waves predicted by the parametric study qualitatively fit the results of the PCB (PCB is a type of pressure sensor manufactured by PCB Piezotronics company) experiments completed in Peking University [36,37], as shown in Fig. 13.

One interesting discovery in the study by Chen *et al*. [72] is the attractor-like character of the parametric resonance in the transitional boundary layer, in the sense that modes with certain frequencies and azimuthal wave numbers will always come into resonance when the amplitudes of the difference/sum modes are compared with the low-frequency mode (which means that the path is close to the resonance route). The saturated state of the second mode seems to dominate the parametric resonance that is probably active in other second-mode-dominated boundary layers (see [73–75]). For those boundary layers, however, the low-frequency waves may be linearly stable and more plausibly arise from either the sideband interactions of the unstable waves or interactions between a pair of oblique unstable waves.

### Transition mechanism in the hypersonic boundary layer

Almost all the experimental studies paid attention to the instability of the second mode [12,50,76]. The transitions were shown to be triggered by the first mode instead of the second mode, which also agrees well with the numerical simulation by Huang *et al*. [77]. Even in the experiments by Casper *et al*. [76] and Stetson and Kimmel [12] under both quiet and noisy conditions, the trace of the second mode could be obtained after the transition. It is necessary to clarify the effects of the second mode in the transition process.

Fasel and co-workers [75] attributed the second mode's decay to the nonlinear resonance of the second mode with its harmonics. However, it is not necessarily the only mechanism. Because the second-mode instabilities are acoustic, and the instabilities may be accompanied with both vortical and dilatational waves, viscous dissipation cannot be neglected. The viscous dissipation function per unit volume $\phi $ can be written as [78,79]:

(1) \begin{equation*} \phi = \mu {\omega ^2} + \left( {\lambda + 2\mu } \right){\vartheta ^2} \end{equation*}

where $\lambda $ is the second coefficient of viscosity by Stokes [80]. The second term in Eq. (1) stands for the dissipation caused by dilatation, which has been considered as a stabilizing effect on the second mode. Let the longitudinal viscosity be $\mu ' = {\rm{\ }}\lambda + 2\mu .$ If Stokes' hypothesis holds, i.e.${\rm{\ \ }}{\mu _b} = {\rm{\ }}0{\rm{\ }}$and $\lambda \ = {\rm{\ }} - 2\mu /3$, then $\mu ' = $$4\mu /3$. In our study, the wall temperature is about 300 K, so $\mu '{\rm{\ }}$was chosen to be 2.06$\mu$—its value in low-pressure N_2_ at *T* = 293 K [81].

**Figure 14.**
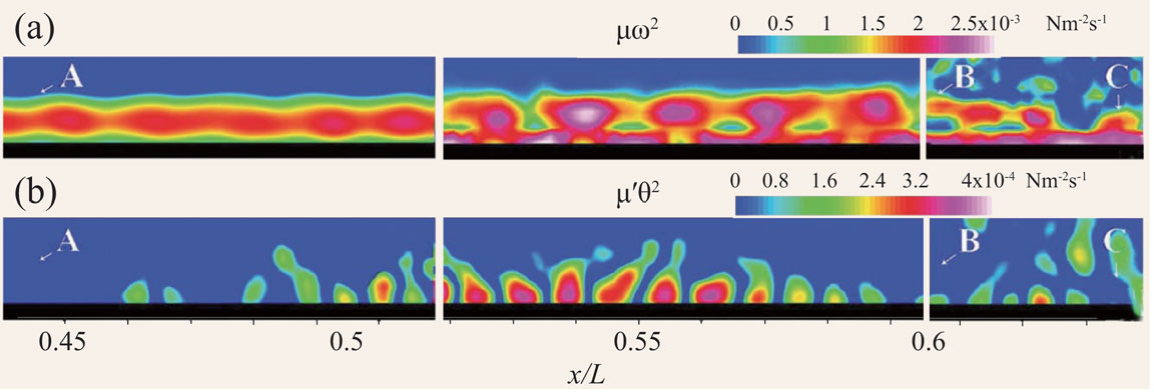
Viscous dissipation induced by (a) vorticity and (b) dilatation. Reprinted with permission from [37]. Copyright American Institute of Aeronautics and Astronautics.

Figure 14 shows the viscous dissipation induced by vorticity (Fig. 14a) and dilatation (Fig. 14b). The viscous dissipation values were extracted from the figure. The dissipation caused by ${\rm{\mu '}}{\vartheta ^2}$ was sampled at the local peak points of the region where $\vartheta \ne {\rm{a}}$.

**Figure 15.**
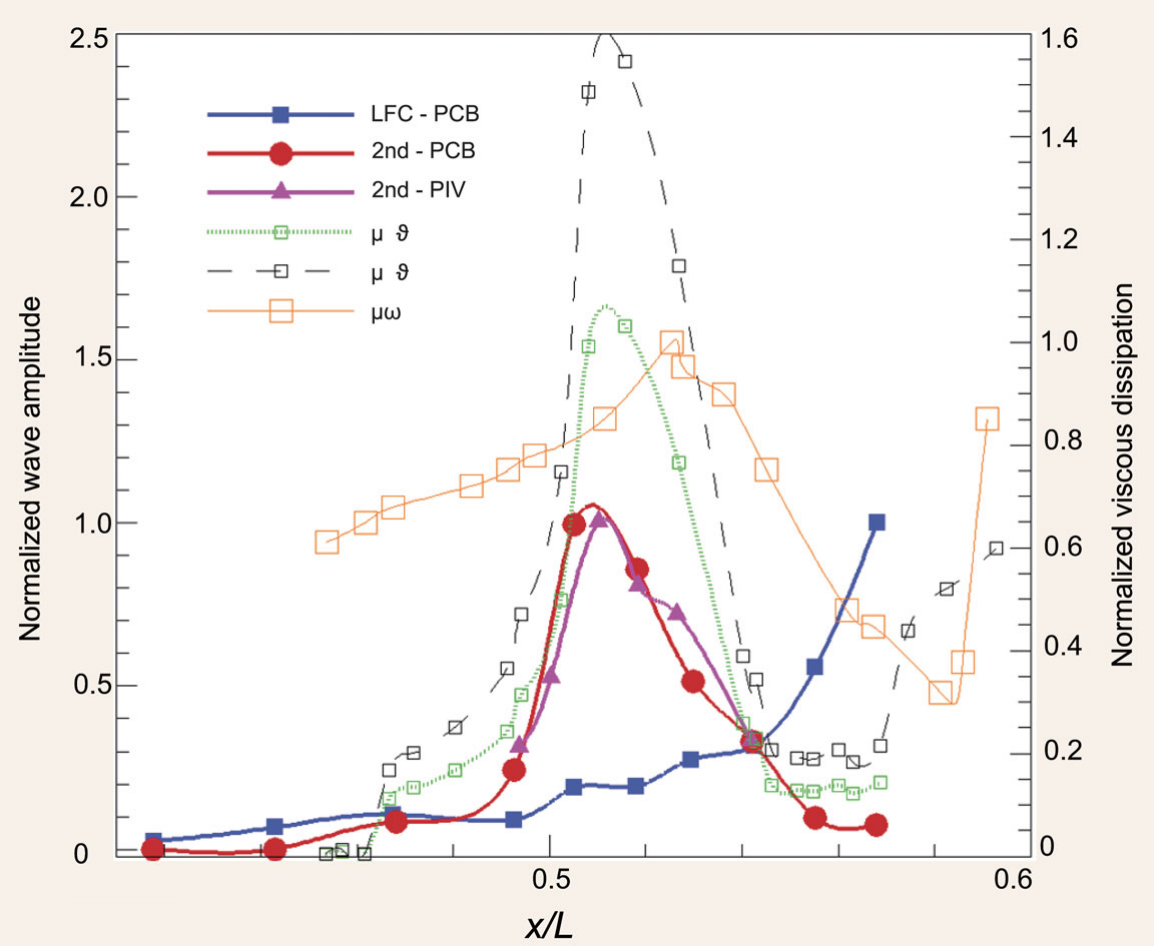
Comparison of wave amplitude and dissipation function by shear and dilatation. Reprinted with permission from [37]. Copyright American Institute of Aeronautics and Astronautics.

**Figure 16.**
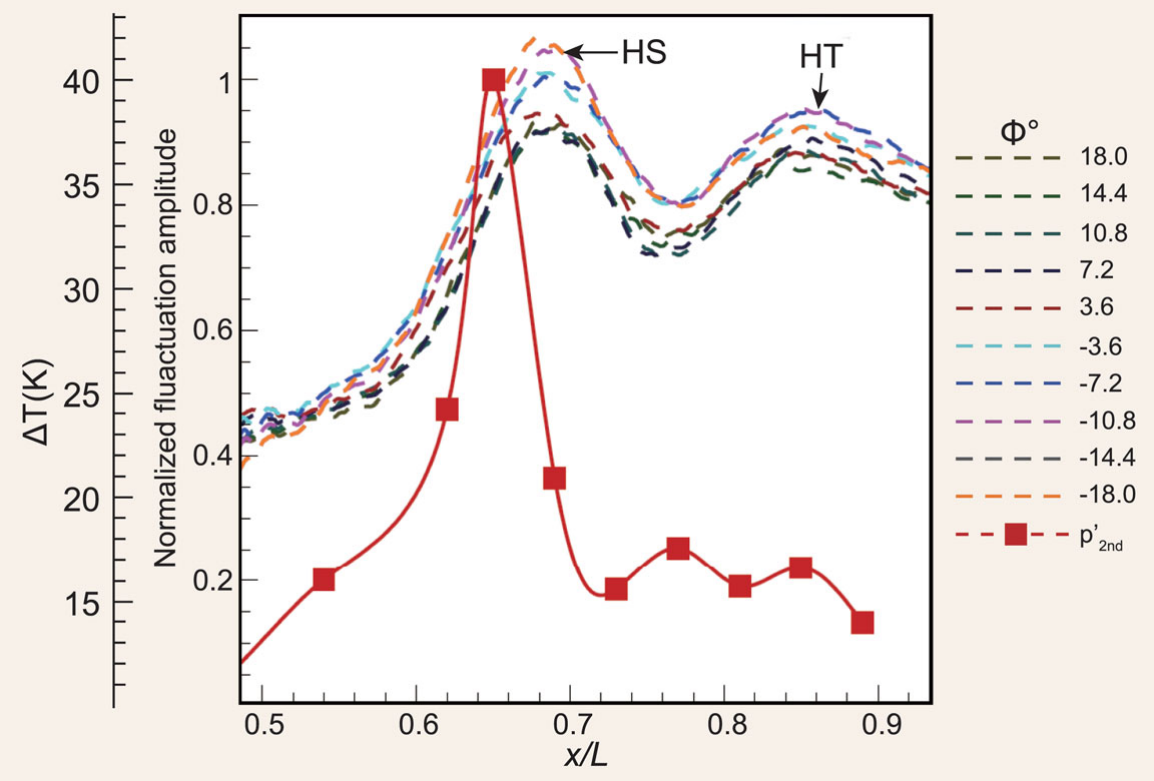
Comparison of surface temperature rise and second-mode-instability evolution based on experiments; Re_unit_ = 9.7 × 10^6^ m^−1^. Reprinted with permission from [73]. Copyright AIP Publishing.

The amplitude of the instability waves and the viscous dissipation can be calculated by combining the simultaneous pressure and PIV measurements. It displays clearly the peak values of ${\rm{\mu }}{{\rm{\omega }}^2}$ and ${\rm{\mu '}}{\vartheta ^2}$, as well as their correlation with the spatial evolutions of low-frequency and second-mode wave amplitude (Fig. 15).

**Figure 17.**
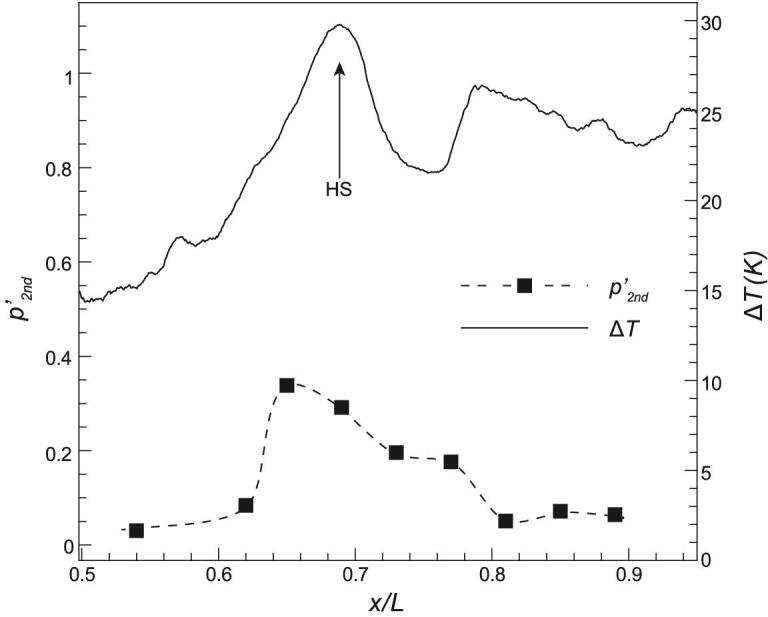
Comparison of surface-temperature rise with second-mode-instability evolution; Re_unit_ = 9.7 × 10^6^ m^−1^. Obtained from TSP results and time-averaged PCB spectra. The subscript 2nd represents the second mode.

The main effect of the second mode lies in aerodynamic heating (Fig. 16) [71]. Recent numerical simulations and experiments have indicated the appearance of an additional peak in heat transfer (denoted herein as HS) within the transitional region and a second rapid growth of heat transfer near the end of transition (denoted herein as HT). The second growth was observed in previous studies [82,83]. Based on direct numerical simulations (DNS), Franko and Lele [84] investigated the transition mechanisms of a Ma = 6 planar hypersonic boundary layer. Three types of such mechanisms were identified: first-mode oblique interaction, second-mode fundamental resonance, and second-mode oblique interaction. For the first-mode oblique interaction mechanism, a distinct overshoot of heat transfer was observed near the end of the transitional region, HT, which was attributed to the generation of streamwise vortices and correspondingly increased wall shear stress. However, for the fundamental resonance dominated by the 2D second mode, an additional peak of heat transfer, HS, appeared where the second mode reached its maximum. The temperature values at HS are slightly higher than those at HT, while the associated skin friction of the former is no more than half of the latter. Horvath *et al*. [85] and Wadhams *et al*. [86] stated that the appearance of HS was attributed to the second-mode evolution, but no physical explanation was given in terms of fundamental thermo-aerodynamics. Sivasubramanian and Fasel [75] used DNS to investigate the laminar-to-turbulent transition in a sharp-cone boundary layer at Ma = 6. They focused on the fundamental resonance mechanism. Streamwise hot streaks were observed before the boundary layer became turbulent. Either spanwise-averaged heat transfer or that along a single streak exhibited both HS and HT in the streamwise direction. The Stanton number at HS was twice that at HT, but the skin friction of the former was only half that of the latter. Using temperature-sensitive paint, Schneider's group [87] observed hot streaks on a Ma 6 flared cone under quiet-flow conditions. The spanwise-averaged temperature exhibited a second rapid growth (HT), which was not quite as strong as that in the midst of the transitional region (HS). PCB piezoelectric pressure sensors were also applied within that region and indicated the appearance of second-mode instability. However, the spatial resolution of the experiments was insufficient to elucidate the relation between the evolution of second-mode instability and the temperature distribution. Schneider's group attributed HS to the nonlinear development of second-mode instability, but no further investigation has been performed on its mechanism. Understanding the physics of second-mode instability is clearly important. Owing to the boundary condition at the sonic line, every reflection of the wave at that line leads to an alternating change between compression and expansion of the fluid. Laurence *et al*. [88,89] experimentally investigated a 1.1-m-long, 7° half-angle cone in a free-piston-driven, reflected-shock wind tunnel under high-entropy conditions. Employing a 1,000 W short-arc Xe lamp together with a camera at frame rates of up to 1 MHz, they obtained schlieren images of typical structures of the second mode. The intensity variation in the image manifested the density variation caused by the inherent compression and expansion process inherent in the second-mode instability. However, the schlieren technique provides only semi-quantitative information because it is difficult to calibrate. As a powerful technique that provides an instantaneous flow field in a plane or a volume, PIV has been successfully applied in experimental investigations of hypersonic flows [36,37,90,91]. By comparing the PIV and PCB sensor measurements, Zhu *et al*. [36,37] found that the evolution of second-mode instability is accompanied by a strong compression and expansion process near the wall.

**Figure 18.**
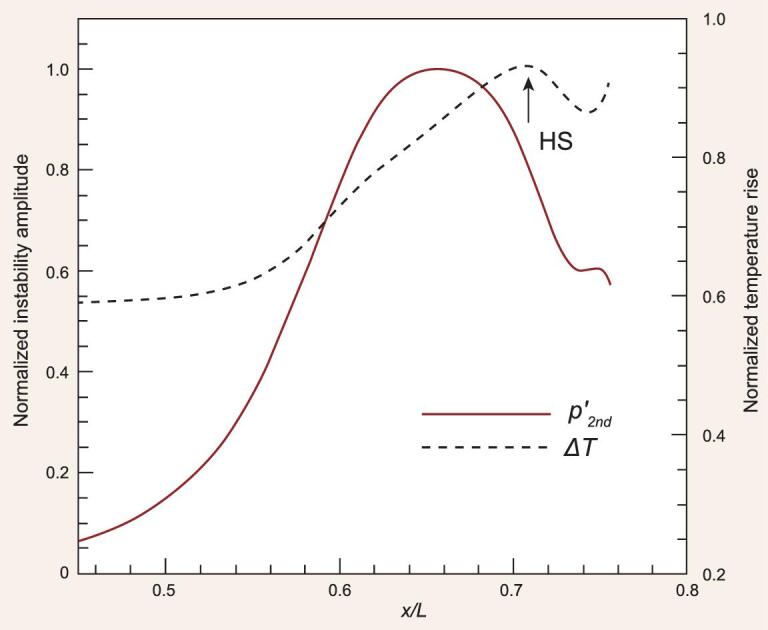
Comparison of the surface-temperature rise and second-mode-instability evolution based on PSE simulations; Re_unit_ = 9.7 × 10^6^ m^−1^. The subscript 2nd represents the second mode. Reprinted with permission from [73]. Copyright AIP Publishing.

**Figure 19.**
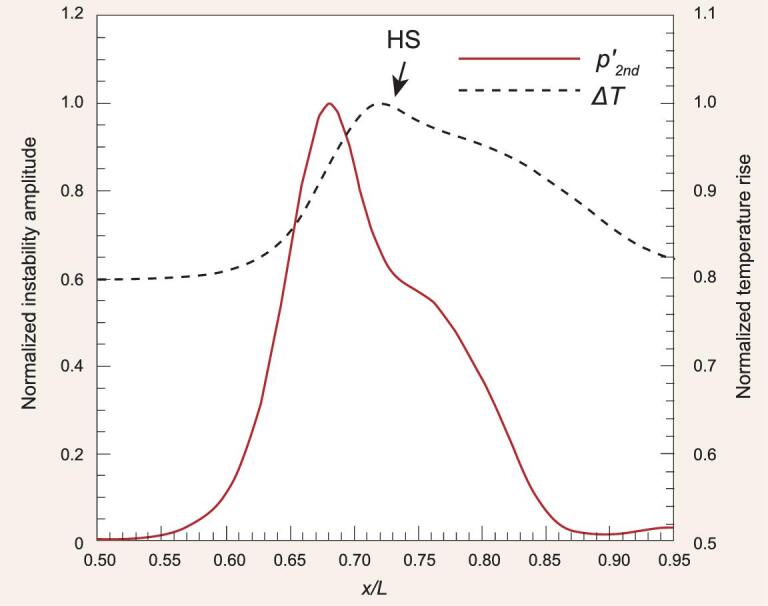
Comparison of surface temperature rise and second-mode-instability evolution based on DNS; Re_unit_ = 9.7 × 10^6^ m^−1^. The subscript 2nd represents the second mode. Reprinted with permission from [73]. Copyright AIP Publishing.

A combined experimental, numerical, and theoretical study of instability modes in a hypersonic boundary layer and their relevance to surface aerodynamic heating is reported in [73]. It confirms the previously observed heat-transfer peak, and a new physical mechanism is proposed to explain this heating impact, which is strongly connected to the evolution of the instability wave. A direct relation between the local heating peak and the viscous dissipation induced by the second mode is examined for the first time. Figure 17 compares the evolution of second-mode instability with the spanwise-average surface-temperature rise. The second-mode amplitudes are normalized to their maximum value. For the highest Reynolds number, the second-mode peak appears at *x*/*L* = 0.65, corresponding to a local peak of temperature at *x*/*L* = 0.68. Such correlations between the second mode and the surface temperature are also observed both in PSE analysis (Fig. 18) and DNS (Fig. 19).

Both the physical and numerical experiments showed that the evolution of the second mode brought about a local peak of surface-temperature HS that shifted slightly downstream. Increasing Re_unit_ promoted the strength of HS, leading to a stronger dissipation of the second mode. The viscous dissipation functions induced by the shear and dilatation processes (denoted as, respectively, Φ_ω_ and Φ_ϑ_) were investigated. In the region HS, the dilatational dissipation Φ_ϑ_ generated by the high-frequency compression and expansion of fluid accompanying the second mode played a dominant role in the formation of HS. Downstream, Φ_ϑ_ decayed as the second mode did, while Φ_ω_ kept on growing, which stimulated a second growth of the surface temperature in the region HT. This is in accordance with Franko and Lele [84]. This new observation is shown to be vitally important for the investigation of transition in the hypersonic boundary layer and is considered as a new principle for aerodynamic heating [92,93].

## CONCLUSION

As discussed above, the second mode is very important for aerodynamic heating and enhances transition. It interacts with the first mode and offers energy to the transition. Therefore, it is essential to stress the important effects of the second mode. However, the transition is controlled by the first mode, as demonstrated by Stetson and Kimmel [12], Bountin *et al*. [59] and Huang *et al*. [77], as well as our recent work [49]. The transition is the Reynolds number dominating phenomenon, which is directly related to the shear stress. The first mode is directly related to the shear stress, while the second mode is related to the compressible parameter, which has strong effects on flow compression and expansion. The flow compression and expansion can directly produce aerodynamic heating and affect the flow shear stress. Nevertheless, compared with the first mode, the effects of the second mode on the shear stress are not significant.

The theory of nonlinear parabolized stability equations (NPSE) shows the detailed interaction between the first mode and the second mode [72], which is in accord with the result obtained from experiments [37]. The parametric study shows that very low-frequency modes are most heavily promoted. In addition, the typical evolution of the low-frequency mode consists of four stages, namely the linear stage, the synchronization stage, the parametric-resonance stage and the full-interaction stage. In the linear stage, the low-frequency mode interacts with the second mode, and a fast-growing difference mode as well as a sum mode is derived. After a quick modification in the synchronization stage, the low-frequency mode enters the third stage, where its growth rate is much larger than before. In the last stage, the low-frequency mode with large amplitude produces a back reaction on the second mode, reducing the amplitude of the second mode.

Improved experimental techniques such as PIV measurement and Rayleigh-scattering visualization show the spatial information of the boundary layer. They lead to the possibility of finding new physics for the study of the hypersonic boundary-layer transition. LST and nonlinear theory (NLT) as well as PSE are powerful tools for the theoretical analysis of such complex fluid flows. DNS data are very useful because they offer all field information that can be used to make up the shortcomings of the experimental data. TSP is a more powerful experimental technique that can be used to predict the all-surface transition region, which satisfies the need for measurement accuracy. Furthermore, a large amount of experimental time has been devoted to hypersonic flow experiments, and time saving has important meanings. For example, one surface transition position prediction at one experimental condition needs just several seconds, which is unthinkable if we only use single-point hot-wire or film measurements.

## PERSPECTIVES

Hypersonic flow transition will be one of the focuses of fluid mechanics in the future, due to the importance of both fundamental research and real application. Basic research will still focus on receptivity; that is to say, what kind of disturbance will enter into the boundary layer or shear layer, generate instability, produce coherent structure and then develop into turbulence. Linear stability, weak nonlinear stability, PSE and NPSE will still play a role in the coming study. Numerical calculation has been shown to be a powerful technique, and will still need to play a role in the study of receptivity. More importantly, an experimental study on this issue is a task of top priority.

In addition to the theoretical and numerical methods, high spatiotemporal resolution experiments are vital to the study of the issue of linear and nonlinear interactions. Developing the method of structural recognition and display based on Lagrange features is still the focus of experimental and numerical research. It is very urgent for a numerical study to develop a strong spatiotemporal coupling calculation method.

The mechanism of transition is still focused on the evolution of the first mode, the second mode and their interactions. More attention should also be paid to the excitation of vibration degree and chemical reaction, as hypersonic flows with Ma > 5 have reached the regime of aerothermochemistry.

The core difficulty of hypersonic transition also lies in research and the development of a feasible engineering transition model. The geometric shape of the flight vehicle is very complex. The flow environment conditions and flight conditions are also different. To establish a universal transition prediction model that satisfies the different and complex flow conditions is still a core task and great challenge.

## Figures and Tables

**Table:** 

Ma	Mach number
*p*′	RMS value of Pitot pressure fluctuation measured by Kulite pressure transducers
*p*_mean_	average value of Pitot pressure fluctuation measured by Kulite pressure transducers
*T*	temperature
*T*_ref_	reference temperature
*R*	universal gas constant
*E*_nr_	activation eneygy for the non-radiative process
*N*	the space amplification factor for a certain disturbance
α_i_	the space amplification rate for a certain disturbance
Φ	viscous dissipation function per unit volume
μ	the first coefficient of viscosity
λ	the second coefficient of viscosity
μ′	the longitudinal viscosity
μ_b_	the coefficient of bulk viscosity
ω	vorticity
θ	divergence
Re_unit_	the unit Reynolds number of the freestream
Φ_ω_	the viscous dissipation functions induced by the shear processes
Φ_ϑ_	the viscous dissipation functions induced by the dilatation processes
